# A Novel Biosorbent From Hardwood Cellulose Nanofibrils Grafted With Poly(*m*-Aminobenzene Sulfonate) for Adsorption of Cr(VI)

**DOI:** 10.3389/fbioe.2021.682070

**Published:** 2021-05-17

**Authors:** Yong Ho Yu, Liangliang An, Jin Ho Bae, Ji Won Heo, Jiansong Chen, Hanseob Jeong, Yong Sik Kim

**Affiliations:** ^1^Department of Paper Science and Engineering, College of Forest and Environmental Sciences, Kangwon National University, Chuncheon, South Korea; ^2^Wood Chemistry Division, National Institute of Forest Science, Seoul, South Korea

**Keywords:** cellulose nanofibrils, hardwood, detoxification, adsorption, Cr(VI), conductive polymer

## Abstract

Cellulose from different lignocellulosic biomass can be used to prepare various materials. In this work, the cellulose nanofibrils were produced from hardwood bleached kraft pulp. Then, a novel biosorbent from cellulose nanofibrils grafted with poly(*m*-aminobenzene sulfonate) (PABS) was prepared for effective detoxification and adsorption of Cr(VI) in an aqueous medium. 6,6-tetramethylpiperidine-1-oxyl (TEMPO)-oxidized cellulose nanofibrils (TOCNF) with a high aspect ratio was used as an adsorbent matrix. PABS, an amine-rich conductive polymer, was grafted onto TOCNF via a successive two-step reaction. The analyses of Fourier transform infrared (FT-IR) spectroscopy and X-ray photoelectron spectroscopy (XPS) confirmed the successful grafting reaction between TOCNF and PABS. The biosorbent from TOCNF-bonded PABS with the nitrogen content of 7.0% was synthesized. It exhibited excellent Cr(VI) adsorption capacity at a solution pH below 3, and almost 100% Cr(VI) can be removed. The adsorption of Cr(VI) on the biosorbent was described by a pseudo-second-order model and obeyed the Langmuir model. The Cr(VI) adsorption capacity of the biosorbent from TOCNF-bonded PABS was almost 10 times higher than that of TOCNF. It was interesting to note that part of Cr(VI) ions had been reduced to Cr(III) during the adsorption process. It indicated that the biosorbent from TOCNF grafted with PABS could detoxify and adsorb Cr(VI) synchronously.

## Graphical Abstract

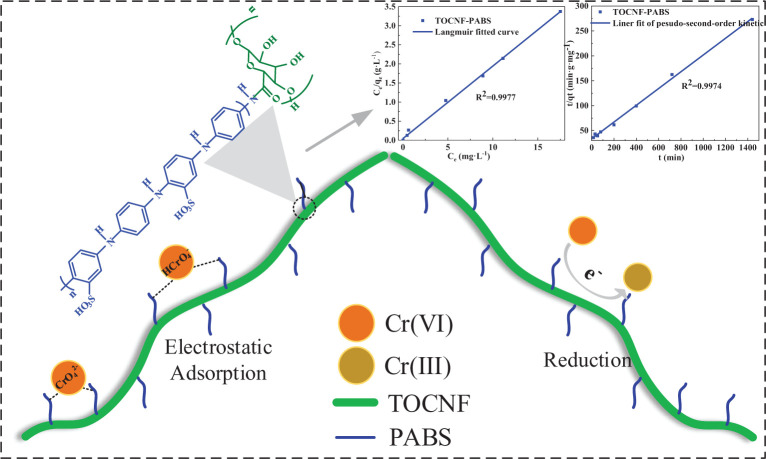

## Introduction

Chromium is a common pollutant for surface and groundwater resources, usually produced from industrial processes, such as tanning, textile, electroplating, steel production, and wood preservation (Mishra and Bharagava, 2016). Chromium generally exists with the trivalent Cr(III) and hexavalent Cr(VI) states. Compared to Cr(III), which is less toxic and can be readily removed in the form of insoluble precipitates, Cr(VI) is the most toxic form, being carcinogenic and mutagenic to living organisms (Chen et al., 2015, 2020). Therefore, Cr(VI) must be substantially removed from wastewater before discharge into the aquatic system. Numerous techniques have been developed to remove Cr(VI) from wastewater, including reduction, precipitation, ion exchange, membrane separation, and adsorption (Jobby et al., 2018). Among these, adsorption is promising and has been widely used due to its easy operation and high efficiency. For example, activated carbon prepared from various matrices has been used as an adsorbent to remove Cr(VI) from an aqueous solution (Wong et al., 2018; Wang P. et al., 2020).

In recent years, biomass-based adsorbents have attracted significant attention as a way to remove Cr(VI) from aqueous solution due to their environmental friendliness, renewability, and biodegradability, such as cellulose (Liang et al., 2020), lignin (Shi et al., 2020), starch (Mohamed and Mahmoud, 2020), and chitosan (Khalil et al., 2020). Cellulose is the most abundant natural polymer on earth and has excellent mechanical and chemical stability properties (An et al., 2019; Liu et al., 2020a; Liu H. et al., 2021; Liu K. et al., 2021). Additionally, cellulose with different sizes and microconformation can be used to produce microcrystalline cellulose, cellulose nanofibrils, and cellulose nanocrystals (Lu et al., 2019; Huang et al., 2020; Liu et al., 2020b; Wang H. et al., 2020; Xu et al., 2021). In particular, cellulose nanofibrils with a high aspect ratio, large surface area, and plentifully available surface hydroxyl groups that can be functionalized to tailor specific properties show great potential as precursors to produce effective adsorbent materials (Zhang et al., 2016; Li et al., 2019; Wang Y. et al., 2020).

Many modifications have been performed for cellulose, including blending and grafting of functional components to increase the active sites of the adsorbent and its uptake capacity for Cr(VI) (Olivera et al., 2016; Jamshaid et al., 2017). The amine group is the most widely explored functional group for enhancing Cr(VI) adsorption owing to its high ability to capture Cr(VI) through electrostatic, reduction/chelating, and hydrogen bonding interactions (Song et al., 2015; Zhang et al., 2020). The amine-/imine-rich conductive polymers with redox flexibility have shown an interesting fact. They can adsorb negatively charged Cr(VI) onto protonated amine/imine groups and convert the highly toxic Cr(VI) to the much less toxic Cr(III) in an aqueous medium (Ding et al., 2018). Research about adsorbents from cellulose cooperating with conductive polymers for removal of Cr(VI) have arisen, including polypyrrole/cellulose composite (Hosseinkhani et al., 2020), polyaniline/cellulose composite (Liu et al., 2013), polyaniline/cellulose nanocomposite (Hosseini and Mousavi, 2021), and polyaniline-coated cellulose (Qiu et al., 2014). For example, Liu et al. (2012) reported an integrated approach for synchronous Cr(VI) detoxification and adsorption with the polyaniline/cellulose fiber composite. Qiu et al. (2014) reported that polyaniline-coated ethyl cellulose greatly removed Cr(VI). Both amine groups of polyaniline and hydroxyl groups of cellulose participated in the Cr(VI) reduction and adsorption. However, these reports only focused on blending or coating cellulose with conductive polymers. There is little research about bio-adsorbents from cellulose covalently bonded conductive polymers to remove Cr(VI).

In this work, a novel biosorbent from cellulose grafted with a conductive polymer was designed. Poly(*m*-aminobenzene sulfonate) (PABS), a sulfonated polyaniline polymer with unique electroactive properties, thermal stability, and water solubility, was synthesized (Bandyopadhyay et al., 2017). 6,6-Tetramethylpiperidine-1-oxyl (TEMPO)-oxidized cellulose nanofibrils (TOCNF) were prepared as an adsorbent matrix. A novel biosorbent from cellulose nanofibrils grafted with conductive polymer (PABS) was fabricated by a successive two-step reaction. PABS-grafted cellulose nanofibrils were characterized by Fourier transform infrared (FT-IR) spectroscopy, X-ray photoelectron spectroscopy (XPS), and elemental analysis. The adsorption of Cr(VI) on the biosorbent in an aqueous medium was performed. The effects of adsorbent dosage, pH, and settling time on the removal amount were studied. Adsorption isotherms and adsorption kinetics were investigated.

## Materials and Methods

### Materials

Hardwood bleached kraft pulp (HWBKP) was provided by Moorim P&P Co., Ltd. (South Korea). Aniline (99.5%), ammonium persulfate (APS, 98%), *m*-aminobenzene sulfonic acid (ABS, 99.0%), sodium thiosulfate anhydride (95%), potassium dichromate (99.5%), 1,5-diphenylcarbazide (DPC, ≥96%), and TEMPO radical (99%) were purchased from Sigma-Aldrich. Acetone (99.5%), dimethylformamide (DMF, 99.5%), oxalyl chloride (98%), hydrogen chloride (35%), ethyl alcohol (99.5%), sulfuric acid (70%), sodium bromide (99%), and sodium hydroxide (98%) were purchased from DaeJung Chemicals & Metals Co., Ltd. (South Korea). Sodium hypochlorite solution (12%) was purchased from YAKURI (Japan). Potassium dichromate (99.5%) was purchased from Kanto Chemical (Japan). These reagents were used without further purification.

### Preparation of PABS

Poly(*m*-aminobenzene sulfonate) was prepared according to a methodology from a previous work (Pradeep et al., 2014). First, 0.865 g of ABS was dissolved in 30 ml of 1 mol⋅L^–1^ HCl solution. Then the mixture solution was stirred at 0°C for 30 min. Then aniline (90.9 μl) was added into the mixture and stirred for another 30 min. Finally, 15 ml of 1 mol⋅L^–1^ APS solution was added into the mixture and stirred at 0°C for 6 h. After the reaction, the mixture was poured into excess acetone. The precipitated solid was filtrated and washed with acetone to remove the unreacted chemicals. The crude PABS was dissolved in 30 mL of deionized water and filtrated to remove insoluble impurities. The soluble PABS was transferred to a dialysis bag [Cellu⋅Sep H1, molecular weight cutoff (MWCO): 1,000, United States] for 72 h. After that, the soluble part was freeze-dried to obtain the purified PABS product.

### Preparation of TOCNF

First, 20 g of HWBKP was disintegrated at 3,000 rpm in a disintegrator (Lorentzen & Wettre 970154, Sweden). This disintegration was operated three times to disintegrate a total of 60 g of HWBKP. Afterward, the fiber suspension (45 g of dried HWBKP) was oxidized using 157.5 ml of NaClO, catalytic TEMPO (0.72 g), and NaBr (0.45 g), according to a methodology developed by Alves et al. (2020). The TEMPO-oxidized cellulose (TOC) was filtered and washed thoroughly with deionized water. Finally, TOCNF were produced by mechanical treatment of 1% TOC using a grinder (MKVA6-2, Masuko Sangyo, Japan) with stone spacing of 150–200 μm for passing 15 times. The prepared TOCNF were kept in a refrigerator for future use.

The morphology of TOCNF was analyzed using an ultra-high-resolution field emission transmission electron microscope (FE-TEM)/energy-dispersive X-ray spectroscope (EDS) (JEM-2100F, JEOL Ltd., Japan) to determine the diameter of TOCNF. Besides, the carboxyl content was determined in duplicate by conductometric titration of the aqueous suspension of TOCNF (acidified to pH 3) with 0.01 mol⋅L^–1^ NaOH. An intrinsic viscosity measurement was conducted in the TOCNF suspension by dissolving it in cupriethylenediamine according to the ISO standard 5351:2010. The degree of polymerization (DP) was estimated using the Mark–Houwink equation with parameters *K* = 0.42 and *a* = 1 (Mendoza et al., 2019).

The yield of TOCNF was found to be almost 100% (no phase separation was observed after mechanical treatment). The average diameter of TOCNF was analyzed to be 7.81 nm (Supplementary Figure 1). Besides, the carboxyl content was determined to be 3.38 mmol⋅g^–1^, and the DP from the intrinsic viscosity was estimated to be 735.

### Preparation of TOCNF Grafted With PABS Copolymer (TOCNF-PABS)

The TOCNF-PABS copolymer was prepared via a two-step reaction, as shown in Supplementary Figure 2. First, an aqueous suspension of TOCNF (0.4 g) was subjected to solvent exchange with DMF by centrifugation at 2,000 rpm three times. Then, TOCNF was dispersed in 50 ml of dried DMF and stirred at 0–5°C for 30 min under N_2_ atmosphere. Next, 1 ml of oxalyl chloride was slowly added into the mixture. The mixture was stirred at 0–5°C for 2 h and then at room temperature for another 2 h. After the reaction, the temperature was raised to 70°C and stirred overnight to remove unreacted oxalyl chloride. Finally, the suspension was poured into deionized water. The TOCNF acyl chloride (TOCNF-Cl) was recovered by centrifugation, and DMF was used to wash unreacted chemicals. Finally, the obtained TOCNF-Cl was dispersed in 50 ml of dried DMF with 300 mg of PABS and stirred at 100°C for 24 h under N_2_ atmosphere. After the reaction, the mixture was poured into excess deionized water. The precipitate was recovered by centrifugation, and water was used to remove unreacted PABS. TOCNF-PABS was freeze-dried and collected.

### Characterization of PABS and Cellulose Samples

The chemical structure analyses of the synthesized PABS and cellulose samples were conducted with an FT-IR spectrophotometer (PerkinElmer Frontier, United States) equipped with an attenuated total reflectance accessory. The spectra were recorded in the wavenumber range of 500–4,000 cm^–1^ at a resolution of 4.0 cm^–1^ (An et al., 2020b).

^1^H nuclear magnetic resonance (^1^H NMR) and gel permeation chromatography (GPC) analyses were performed for a more detailed chemical structure analysis of PABS. ^1^H NMR was measured using a 600-MHz Fourier transform nuclear magnetic resonance (FT-NMR) (Bruker Avance Neo 600, Germany). PABS (10 mg) was directly dissolved in 0.5 ml D_2_O (99.96%, Sigma-Aldrich) to record the ^1^H NMR spectrum. The molecular weight was analyzed by GPC (Shimadzu 20A, Japan) equipped with PLgel columns (PLgel 5 μm mixed-C and mixed-D and PLgel 3 μm mixed-E). PABS was dissolved in DMF containing 0.1% LiBr to determine its molecular weight (Youe et al., 2018).

The contents of different elements (C, H, N, and S) of the synthesized PABS and cellulose samples were determined using an elemental analyzer (Eurovector EA3000, Italy) with a thermal conductivity detector. The surface element analysis of cellulose samples was measured by XPS (Thermo Scientific, United Kingdom) (An et al., 2020a).

### Hexavalent Chromium Adsorption Experiments

A Cr(VI) solution with a concentration of 2.5 mg⋅L^–1^ was prepared from potassium dichromate. Next, 30 ml of the Cr(VI) solution was mixed with TOCNF (10–180 mg) and TOCNF-PABS (10–120 mg) at different pH values (ranging 1–11) in a triangular flask and shaken in a shaker (Serker II, VISION Scientific, South Korea) for different times (0–24 h) at 25°C. After adsorption, filtration was performed by using a polytetrafluoroethylene (PTFE) filter paper (0.45 μm), and the filtrate was collected. The DPC method was used for Cr(VI) determination, according to the methodology from a previous work (Seo et al., 2019). To 20 ml of the filtrate were added 0.1 ml of concentrated nitric acid and 1.2 ml of DPC solution. The residual Cr(VI) of the filtrate was analyzed using ultraviolet-visible (UV-Vis) spectroscopy (X-ma 3000, Human Corporation, South Korea) at 540 nm. An inductively coupled plasma optical emission spectrometer (ICP-OES, Agilent, United States) was used to determine the total Cr [Cr(VI)+ Cr(III)] concentration of residual solution after adsorption, and the Cr(III) concentration was obtained by the difference method according to Eq. (1). The adsorbed amount (*Q*) and the adsorption efficiency (*E*) of Cr(VI) were calculated according to the Eqs. (2, 3).

(1) $$C_{f\,3}\,\left(\text{mg}\cdot L^{-1}\right)=C_{f\,\left(3+6\right)}-C_{f\,6}$$

(2) $$\text{Q}\,\left(\text{mg}\cdot {\text{g}}^{-1}\right)=\left({\text{C}}_{i}-{\text{C}}_{f\,6}\right)\times \text{V}/\text{M}$$

(3) $$\text{E}\left(\%\right)=\left[\left({\text{C}}_{i}-{\text{C}}_{f}\right)/{\text{C}}_{i}\right]\times 100$$

where C*_*f3*_* is the Cr(III) concentration of the residual solution after adsorption; C*_*f*_*_(3__+__6)_ is the total Cr [Cr(VI) + Cr(III)] concentration of the residual solution after adsorption; C*_*f*_*_6_ is the final Cr(VI) concentrations of the solution after adsorption; C*_*i*_* is the initial Cr(VI) concentration of the solution; *V* is the volume (L) of the Cr(VI) solution; and *M* is the weight (g) of the adsorbent.

## Results and Discussion

### Characterization of PABS

The chemical structure analysis of PABS copolymer was performed by FT-IR, ^1^H NMR, elemental content, and molecular weight. The ^1^H NMR and FT-IR spectra were illustrated in Supplementary Figure 3. The protons in both aromatic ring and amine were confirmed to be around 7.5–8.0 ppm in ^1^H NMR analysis (Zhao et al., 2004). Furthermore, a series of characteristic PABS peaks can be observed in FT-IR analysis. The bands at 3,200 and 3,056 cm^–1^ were attributed to –NH– stretching and aromatic C–H stretching, respectively. The absorption peaks at 1,600 and 1,411 cm^–1^ were assigned to the vibrations of aromatic rings plus C–N stretching, and a peak around 1,148 cm^–1^ was assigned to S = O stretching (Rao and Sathyanarayana, 2002; Mu, 2005). Additionally, the peaks at 864, 702, and 583 cm^–1^ were assigned to C–H out-of-plane bending vibrations of benzene rings, S–O stretching, and S–C stretching, respectively (Pradeep et al., 2014). Therefore, the chemical structure of PABS can be confirmed through the results of ^1^H NMR and FT-IR analyses.

The molecular weight and element content of PABS were analyzed, as shown in Supplementary Table 1. PABS exhibited a weight-average molecular weight of 18,000 g⋅mol^–1^, a number-average molecular weight of 15,900 g⋅mol^–1^, and a polydispersity index of 1.13. In addition, the elemental contents of C, H, N, O, and S of PABS were 40.1, 3.5, 8.2, 36, and 12.2%, respectively. It was found that the contents of N and S were relatively high due to the presence of amine and a sulfonic group of PABS. Overall, it was concluded that PABS was successfully prepared according to chemical structure analysis (^1^H NMR and FT-IR), molecular weight distribution, and elemental analysis.

### Characterization of TOCNF, TOCNF-Cl, and TOCNF-PABS

(TEMPO)-oxidized cellulose nanofibrils used for the synthesis of TOCNF-PABS was prepared by mechanically treating TEMPO-oxidized HWBKP. TOCNF-PABS was prepared through a two-step synthetic route (Supplementary Figure 2). First, the carboxyl group of TOCNF was substituted with an acyl chloride using an excess amount of oxalyl chloride, and then PABS was grafted onto TOCNF, finally to prepare the TOCNF-PABS. Table 1 shows the yields and elemental analysis results of TOCNF, TOCNF-Cl, and TOCNF-PABS. After the reaction, the yield increased by 26.35%, and the element contents of N (7.0%) and S (3.7%) appeared, indicating that PABS was successfully copolymerized with TOCNF-Cl.

**TABLE 1:** Yields and elemental contents of TOCNF, TOCNF-Cl, and TOCNF-PABS.

**Sample**	**Yield (mg)**	**Elemental content (%)**				
		**C**	**H**	**N**	**O**	**S**
TOCNF	NA	37.9	6.0	0	56.1	0
TOCNF-Cl	400.3	40.5	5.8	0	53.7	0
TOCNF-PABS	505.4	49.1	6.4	7.0	33.8	3.7

*NA, not available.*

**FIGURE 1:**
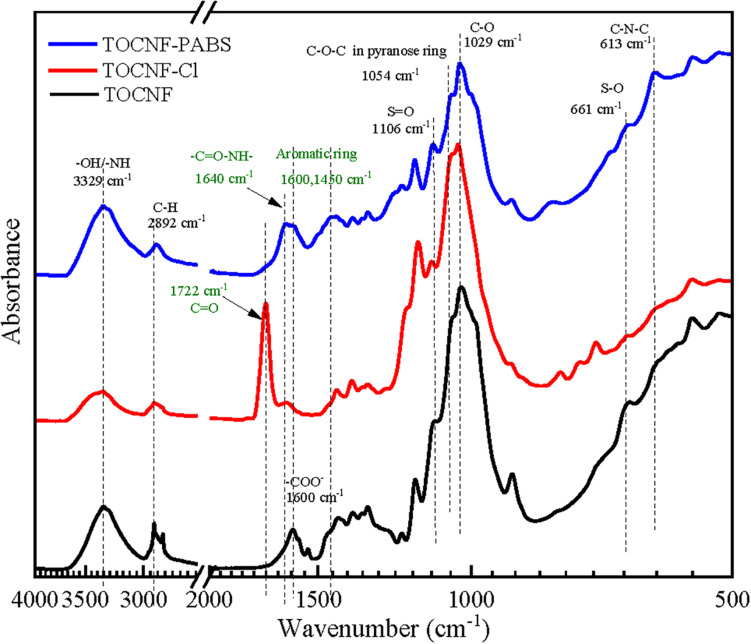
Fourier transform infrared (FT-IR) spectra of TOCNF, TOCNF-Cl, and TOCNF-PABS.

The chemical structure of TOCNF-PABS was analyzed by FT-IR and XPS. Figure 1 exhibited the FT-IR spectra of TOCNF, TOCNF-Cl, and TOCNF-PABS. The typical band at 1,600 cm^–1^ attributed to –COONa stretching was observed in TOCNF (Yuan et al., 2019). After reaction with oxalyl chloride, the carboxyl group of TOCNF was converted to acyl chloride, and a new band at 1,722 cm^–1^ attributed to –COCl stretching appeared, indicating that the carboxyl group was successfully acyl chlorinated. A clear band shifting from 1,722 to 1,640 cm^–1^ was observed in the spectrum of TOCNF-PABS. It is due to –COCl in TOCNF-Cl reacting with –NH_2_ in PABS to form an acylamide (–C = O–NH–), which usually resulted in a peak around 1,640 cm^–1^ (Zhao et al., 2004). It is the primary proof to indicate the successful grafting reaction of PABS onto TOCNF. Additionally, specific functional groups belonging to PABS were identified as follows: the increase in peaks at around 3,329 cm^–1^ was assigned to –OH plus –NH stretching; the peak at 1,600 and 1,450 cm^–1^ were assigned to aromatic ring stretching (Mu, 2005); the peak at 1,106 cm^–1^ was assigned to S = O stretching. Additionally, weak peaks at 661 and 613 cm^–1^ assigned to S–O stretching and C–N–C stretching, respectively, appeared in the spectrum of TOCNF-PABS (Pradeep et al., 2014).

**FIGURE 2:**
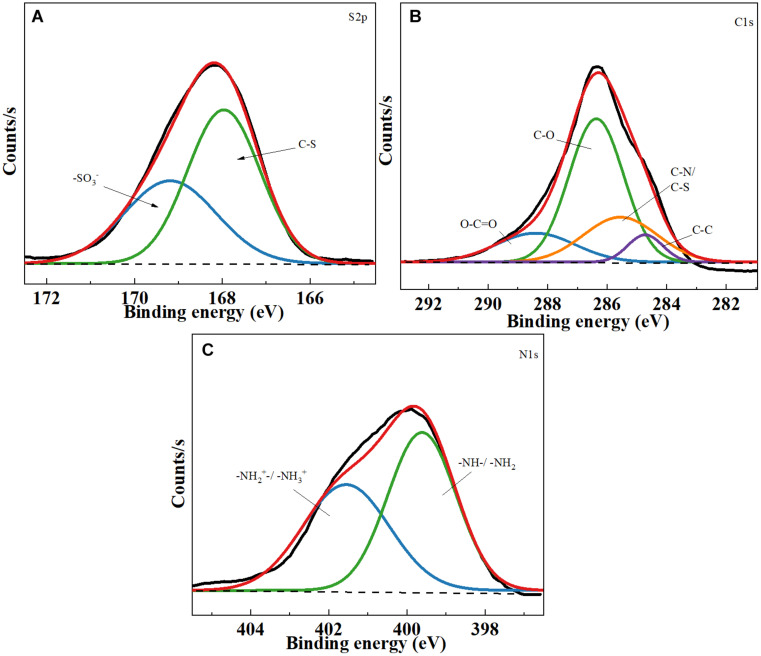
X-ray photoelectron spectroscopy (XPS) spectra of TOCNF-PABS. **(A)** S2p core-level spectra of TOCNF-PABS; **(B)** C1s core-level spectra of TOCNF-PABS; **(C)** N1s core-level spectra of TOCNF-PABS.

Figure 2 shows the XPS survey spectra of TOCNF-PABS. As shown in Figure 2A, the S_2p_ signal was deconvoluted into two components at 168.1 and 169.9 eV, which correspond to the S–O and S–C bonding of PABS, respectively (Huang and Xu, 2010). On the other hand, The C_1s_ peak was deconvoluted into four components at 284.8, 285.8, 286.3, and 289.0 eV that account for the C–C, C–S/C–N, C–O, and O–C = O bonding, respectively (Figure 2B; Liu et al., 2010; Tu et al., 2014). Among these, the C–S/C–N bonding might have originated from PABS. Similarly, the N_1s_ spectrum also detects two peaks, 399.6 eV belonging to the –NH– bonding and 401.4 eV for quaternary ammonium bonding (Figure 2C; Abdulla et al., 2015). All the XPS results also indicated the successful grafting reaction of PABS onto TOCNF. Therefore, considering the above chemical structure analyses, it could be concluded that TOCNF-PABS was successfully prepared through a two-step synthetic route.

### Cr(VI) Adsorption of TOCNF and TOCNF-PABS

**FIGURE 3:**
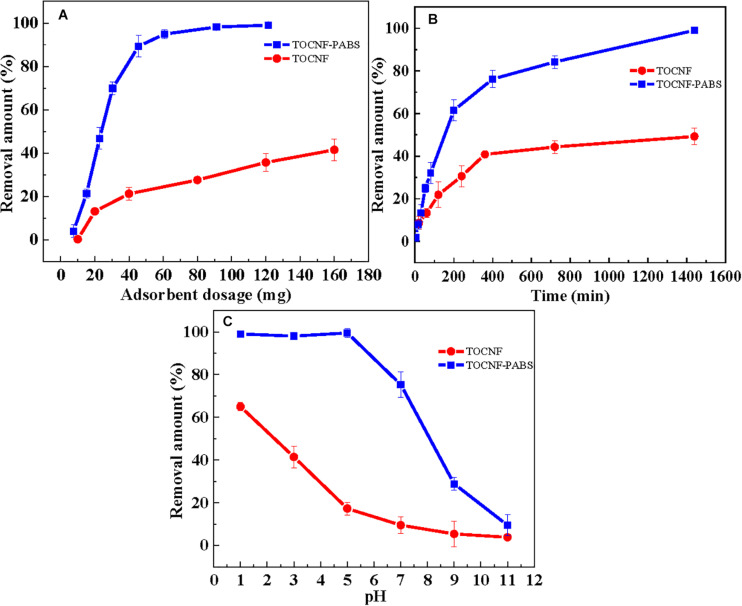
Effect of adsorbent dosage **(A)**, adsorption time **(B)**, and pH **(C)** on the removal amount of Cr(VI).

The amount of adsorbent is an important factor as it directly determines the efficiency of TOCNF-PABS for Cr(VI) adsorption. The effect of TOCNF and TOCNF-PABS dosages on Cr(VI) removal is shown in Figure 3A. The Cr(VI) adsorption was performed using different adsorbent dosages (10–180 mg) at pH 3 for 24 h. As observed, the removal amount increased with the increase of adsorbent dosage, which could be attributed to the increased surface area and presence of more active sites. It was observed that the removal amount increased very rapidly when the amount of TOCNF-PABS was increased from 10 to 60 mg, and the removal amount reached almost 100% for 60 mg. Whereas in the case of TOCNF, even when 160 mg was added, the removal amount was found to be about 40%.

Figure 3B shows the effect of settling time in the range of 0–1,440 min on the removal amount of Cr(VI) for TOCNF (160 mg) and TOCNF-PABS (120 mg). At a settling time ranging from 0 to 400 min, the removal amount of Cr(VI) was rapidly increased to around 80% for TOCNF-PABS, and then, it slowly increased to 100% from 400 to 1,400 min. However, after reaching 40% in 400 min, there was no significant increase in the removal amount of Cr(VI) for TOCNF. This indicated that adsorption equilibrium was attained for both absorbents. In comparison, even though a relatively small amount of TOCNF-PABS was used, a fast adsorption rate was observed for TOCNF-PABS compared to that for TOCNF.

**FIGURE 4:**
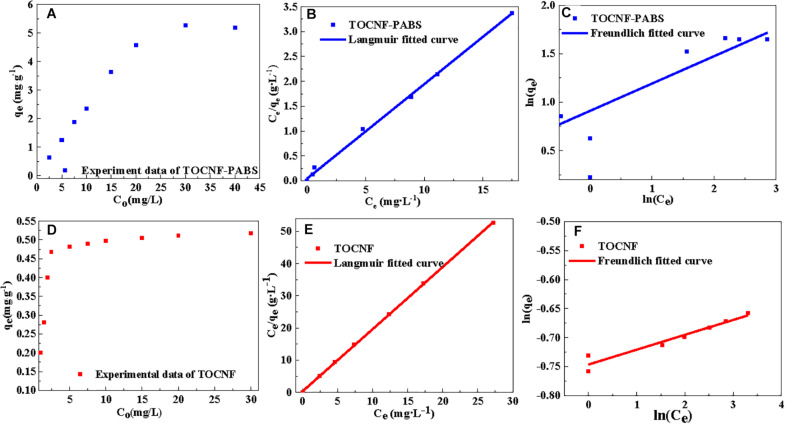
Linear fitting curves of isotherm models (Langmuir and Freundlich models) for the adsorption of Cr(VI) onto TOCNF and TOCNF-PABS. **(A)** Effect of different initial Cr(VI) concentration on the adsorption capacity of TOCNF-PABS; **(B)** Linear fitting curve of Langmuir model for the adsorption of Cr(VI) onto TOCNF-PABS; **(C)** Linear fitting curve of Freundlich model for the adsorption of Cr(VI) onto TOCNF-PABS; **(D)** Effect of different initial Cr(VI) concentration on the adsorption capacity of TOCNF; **(E)** Linear fitting curve of Langmuir model for the adsorption of Cr(VI) onto TOCNF; **(F)** Linear fitting curve of Freundlich model for the adsorption of Cr(VI) onto TOCNF.

The pH is one of the most critical factors affecting the adsorption capacity because it can affect the absorbent’s charge density and the adsorbate’s present state. Usually, the Cr(VI) removal amount was known to be rapidly increased when pH decreased from 8.0 to 2.0. The effect of pH on the removal amount of Cr(VI) is shown in Figure 3C. As observed, the removal amount of Cr(VI) decreased rapidly as the solution pH increased from 3 to 11 when TOCNF-PABS (120 mg) and TOCNF (160 mg) were used. However, the removal amount of Cr(VI) was almost 100% for TOCNF-PABS in the pH range of 1–3, but it decreased from 60% to 40% for TOCNF as the pH increased from 1 to 3. To our knowledge, Cr(VI) ions are known to exist in the form of CrO_4_^2–^ species when the pH was higher than 6 and of HCrO_4_^–^ and Cr_2_O_7_^2–^ species when the pH was between 2 and 6 (Zhao et al., 2015; Liang et al., 2020). Meanwhile, in the low pH range of 1–3, amino groups of TOCNF-PABS were extensively protonated to form either –NH_3_^+^ or –NH_2_^+^– groups, resulting in strong electrostatic attraction with negatively charged Cr anion species (Figure 4). And this caused an increase in Cr(VI) adsorption in the low pH range of 1–3. Likewise, the carboxylic acid group of TOCNF was extensively protonated to form the COOH_2_^+^ cation group, which also adsorbed Cr anion species by strong electrostatic attraction. In contrast, when pH increased from 3 to 11, both sulfonic groups of TOCNF-PABS and carboxylic acid of TOCNF were extensively deprotonated to form SO_3_^–^ and COO^–^ anion groups, respectively, causing strong electrostatic repulsion with negatively charged Cr anion species. So the Cr(VI) removal efficiency fell rapidly. Notably, the Cr(VI) removal efficiency was approximately 100% at pH 3 with TOCNF-PABS, so the adsorption experiments were performed at pH 3.

Additionally, Cr(III) was observed in the residual solution after adsorption. When the pH value was 7, the Cr(VI) removal amount was 57.21%, and the remaining amount of Cr(VI) in solution was 24.66%; then 18.13% Cr(III) was observed in the residual solution. The existence of Cr(III) in the solution indicated that part of Cr(VI) could have been reduced to Cr(III) by TOCNF-PABS during the adsorption process. Some previous works have pointed out that amine groups with high redox potential can reduce Cr(VI) to Cr(III). Part of Cr(III) was released into the aqueous phase owing to the electronic repulsion between the positively charged amine groups and the Cr(III) (Liu et al., 2012, 2013). These results suggested that the biosorbent from TOCNF-bonded PABS could synchronously detoxify and adsorb Cr(VI).

### Cr(VI) Adsorption Isotherms and Adsorption Kinetics

To evaluate Cr(VI) adsorption isotherms, different initial Cr(VI) concentration in the range 1–60 mg⋅L^–1^ was performed on the adsorption for TOCNF (160 mg) and TOCNF-PABS (120 mg) at pH 3 for 24 h. Langmuir and Freundlich adsorption isotherm models were used to fit the adsorption isotherms (Wang et al., 2018). The linear Langmuir and Freundlich equations are given in Eqs. (4, 5), respectively.

(4) $$\frac{C_{e}}{q_{e}}=\frac{C_{e}}{q_{m}}+\frac{1}{q_{m}\,k_{L}}$$

(5) $$\ln q_{e}=\ln k_{F}+\frac{\ln C_{e}}{n}$$

where *q*_*e*_ is the equilibrium adsorption capacity (mg⋅g^–1^), *C*_*e*_ is the concentration of adsorbate at equilibrium (mg⋅L^–1^), *q*_*m*_ is the maximum adsorption capacity (mg⋅g^–1^), *k*_*L*_ is the Langmuir constant, and *k*_*F*_ and *n* are Freundlich constants.

Figures 4A,D showed the effect of the initial concentration of Cr(VI) on the adsorption capacity of TOCNF and TOCNF-PABS, respectively. With the increase of the initial concentration of Cr(VI), the adsorption capacities of both adsorbents were increased and then gradually slowed down from spectra. The maximum adsorption capacities (5.277 mg⋅g^–1^ for TOCNF-PABS and 0.518 mg⋅g^–1^ for TOCNF) were derived from the experimental data. This result indicated that TOCNF-PABS had better Cr(VI) adsorption capacity than TOCNF. Figures 4B,C,E,F show the results of fitting the experimental data to the Langmuir and Freundlich isotherm models. The relevant linear equation parameters are listed in Table 2. The Langmuir isotherm model fitted well the experimental data for the adsorption of Cr(VI) on both TOCNF and TOCNF-PABS, as revealed by the high correlation coefficients (*R*^2^), which were 0.9998 and 0.9977, respectively, whereas for the Freundlich isotherm model, smaller *R*^2^ values were obtained. The maximum adsorption capacity values of Cr(VI) from the Langmuir isotherm models were 5.263 mg⋅g^–1^ for TOCNF-PABS and 0.518 mg⋅g^–1^ for TOCNF.

**TABLE 2:** Characteristic parameters of Langmuir and Freundlich models for the adsorption of Cr(VI) onto TOCNF and TOCNF-PABS.

**Samples**	**Langmuir isotherm**			**Freundlich isotherm**		
	***R*^2^**	***k*_*L*_ (L⋅mg^–1^)**	***q*_*m*_ (mg⋅g^–1^)**	***R*^2^**	***k*_*F*_ (mg⋅g^–1^)**	***n***
TOCNF	0.9998	6.349	0.518	0.9427	0.474	39.00
TOCNF-PABS	0.9977	4.317	5.263	0.5423	2.485	3.546

The adsorption kinetics were determined based on the effect of the adsorption time on the adsorption capacity. The adsorption was performed using TOCNF (160 mg) and TOCNF-PABS (120 mg) with 30 ml Cr(VI) solution (30 mg⋅L^–1^) at pH 3 for 0–24 h. The common mathematical models, pseudo-first-order and pseudo-second-order kinetic models, were used to fit the adsorption kinetics data (Wang et al., 2018). The linear pseudo-first-order and pseudo-second-order kinetic equations are given in Eqs. (6, 7), respectively.

(6) $$\ln\left(q_{e}-q_{t}\right)=\ln q_{e}-k_{1}\,t$$

(7) $$\frac{t}{q_{t}}=\frac{1}{k_{2}\,q_{e}^{2}}+\frac{t}{q_{e}}$$

where *q*_*e*_ is the equilibrium adsorption capacity (mg⋅g^–1^), *q*_*t*_ is the adsorption capacity (mg⋅g^–1^) at time *t* (min), *k*_1_ and *k*_2_ are the kinetics rate constants for pseudo-first-order and pseudo-second-order kinetic models, respectively.

**FIGURE 5:**
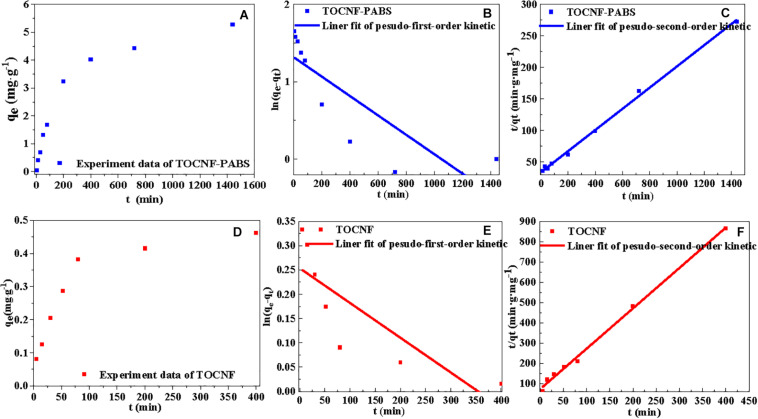
Linear fitting curves of isotherm models (pseudo-first-order and pseudo-second-order kinetic models) for the adsorption of Cr(VI) onto TOCNF and TOCNF-PABS. **(A)** Effect of the adsorption time on the adsorption capacity of TOCNF-PABS; **(B)** Linear fitting curve of pseudo-first-order kinetic model for the adsorption of Cr(VI) onto TOCNF-PABS; **(C)** Linear fitting curve of pseudo-second-order kinetic model for the adsorption of Cr(VI) onto TOCNF-PABS; **(D)** Effect of the adsorption time on the adsorption capacity of TOCNF; **(E)** Linear fitting curve of pseudo-first-order kinetic model for the adsorption of Cr(VI) onto TOCNF; **(F)** Linear fitting curve of pseudo-second-order kinetic model for the adsorption of Cr(VI) onto TOCNF.

Based on the experimental data obtained from the effect of settling time on the removal amount, the pseudo-first-order and pseudo-second-order kinetic models were used to fit the data. The results are presented in Figure 5. The characteristic kinetic equation parameters are listed in Table 3. Figures 5A,D show the effect of settling time on the adsorption capacity of TOCNF-PABS and TOCNF, respectively. With the prolonging of the settling time, the adsorption capacity was increased, and the maximum adsorption capacity was reached. As clearly observed from Table 3, the values of the correlation coefficient *R*^2^ for the pseudo-second-order kinetic models were slightly higher than those of the pseudo-first-order kinetic models and close to 1. Additionally, the maximum capacity values from the pseudo-second-order kinetic models were close to the adsorption experiment data’s values. Therefore, these results indicated that the adsorption of Cr(VI) ions on both TOCNF and TOCNF-PABS followed pseudo-second-order kinetics.

**TABLE 3:** Characteristic parameters of kinetic equations for the adsorption of Cr(VI) onto TOCNF and TOCNF-PABS.

**Samples**	**Pseudo-first-order kinetics**			**Pseudo-second-order kinetics**			
	***R*^2^**	***K*_1_ (min^–1^)**	***q*_*e.cal*_ (mg⋅g^–1^)**	***R*^2^**	***K*_2_ (g⋅mg^–1^⋅min^–1^)**	***q*_*e.cal*_ (mg⋅g^–1^)**	***q*_*e.exp*_ (mg⋅g^–1^)**
TOCNF	0.6896	7.2⋅10^–4^	1.289	0.9967	5.4⋅10^–2^	0.502	0.462
TOCNF-PABS	0.6862	1.3⋅10^–3^	3.741	0.9974	8.4⋅10^–4^	5.949	5.277

**FIGURE 6:**
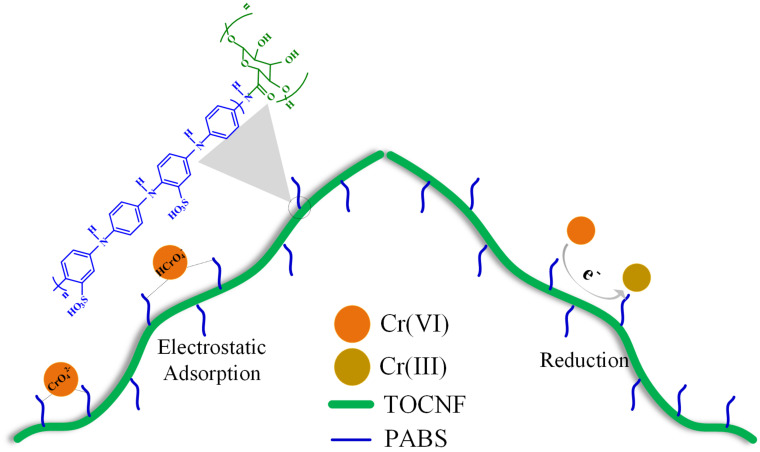
Hypothetical adsorption mechanism of Cr(VI) onto TOCNF-PABS.

As shown in Figure 6, after a two-step reaction, an amine-rich biosorbent was prepared from cellulose nanofibrils and PABS. PABS was dotted on the cellulose chain to form a series of effective adsorption sites. When the solution had a low pH range, Cr(VI) ions existed in the form of negatively charged species, including CrO_4_^2–^, HCrO_4_^–^, and Cr_2_O_7_^2–^. Meanwhile, the amine groups of TOCNF-PABS were extensively protonated to form either –NH_3_^+^ or –NH_2_^+^– groups. It consequently triggered a strong electrostatic attraction between negatively charged Cr anion species and positively charged amine groups. And the electrostatic attraction was the main adsorption attraction for Cr(VI) onto a TOCNF-PABS in the low pH range of 1–3. Additionally, when Cr(VI) was adsorbed onto the biosorbent surface, the amine groups with high redox potential can reduce Cr(VI) to Cr(III). Part of Cr(III) ions was released into the aqueous phase owing to the electronic repulsion between the positively charged groups and the Cr(III). Therefore, the biosorbent from TOCNF grafted with PABS could detoxify and adsorb Cr(VI) synchronously.

## Conclusion

A novel biosorbent from hardwood cellulose and a conductive polymer was designed. The high-aspect-ratio cellulose nanofibrils were grafted with PABS via a two-step reaction. FT-IR and XPS analyses confirmed the successful grafting reaction between TOCNF and PABS. And a high nitrogen content (7%) was observed in TOCNF-PABS, which indicated rich amine groups in TOCNF-PABS. The introduction of effective adsorption sites (amine groups) endues TOCNF-PABS with excellent adsorption capacity. The maximum adsorption capacity was 5.263 mg⋅g^–1^ from the Langmuir model, a significant improvement compared to TOCNF with an adsorption capacity of 0.518 mg⋅g^–1^. Additionally, it has been observed that part of Cr(VI) ions was reduced to Cr(III) during the adsorption process. It indicated that the biosorbent from TOCNF grafted with PABS possessed not only effective adsorption capacity but also detoxification ability for Cr(VI) in an aqueous medium.

## Data Availability Statement

The original contributions presented in the study are included in the article/Supplementary Material, further inquiries can be directed to the corresponding author/s.

## Author Contributions

YY, LA, and YK: idea and experimental designing, and writing – original draft. YY, JB, LA, HJ, and YK: investigation. YK: supervision. YY, LA, JB, JH, JC, and YK: writing – reviewing and editing. All authors contributed to the article and approved the submitted version.

## Conflict of Interest

The authors declare that the research was conducted in the absence of any commercial or financial relationships that could be construed as a potential conflict of interest.
