# Supplementary material for: A Novel Biosorbent From Hardwood Cellulose Nanofibrils Grafted With Poly(m-Aminobenzene Sulfonate) for Adsorption of Cr(VI)
Source: Front Bioeng Biotechnol. 2021 May 17;9:682070. doi: 10.3389/fbioe.2021.682070 (PMC8166254; doi:10.3389/fbioe.2021.682070)
Supplement: Supplementary file 1 [file Table_1.DOCX]

Supplementary Material

Fig. S1. Morphology of TOCNF before (a) and after (b) high pressure homogenization.

Fig. S2. Synthetic schemes for the preparation of PABS and TOCNF-PABS.

Fig. S3. ^1^H HMR (a) and FT-IR (b) spectra of PABS.

Table S1. Molecular weight and elemental content of PABS.

| Sample | Molecular weight | | | Elemental content (%) | | | | |
| --- | --- | --- | --- | --- | --- | --- | --- | --- |
|  | M_w_ (g·mol^-1^) | M_n_ (g·mol^-1^) | PDI | C | H | N | O | S |
| PABS | 18000 | 15900 | 1.13 | 40.1 | 3.5 | 8.2 | 36.0 | 12.2 |
